# Utility of Ischemia-Modified Albumin as a Biomarker for Acute Appendicitis: A Systematic Review and Meta-Analysis

**DOI:** 10.3390/jcm12175486

**Published:** 2023-08-24

**Authors:** Apoorv Singh, Zenon Pogorelić, Aniket Agrawal, Carlos Martin Llorente Muñoz, Deepika Kainth, Ajay Verma, Bibekanand Jindal, Sandeep Agarwala, Sachit Anand

**Affiliations:** 1 Department of Pediatric Surgery, All India Institute of Medical Sciences, New Delhi 110029, India; dr.singhapoorv@gmail.com (A.S.); talk2ajayverma@gmail.com (A.V.); sandpagr@hotmail.com (S.A.); 2 Department of Surgery, School of Medicine, University of Split, 21000 Split, Croatia; zpogorelic@gmail.com; 3 Department of Pediatric Surgery, University Hospital of Split, 21000 Split, Croatia; 4 Department of Pediatric Surgery, Kokilaben Dhirubhai Ambani Hospital, Mumbai 400053, India; aniketagrawal.24967@gmail.com; 5 Surgical Clinic Medix-Muñoz, 28000 Madrid, Spain; llorentecm@gmail.com; 6 Division of Neonatology, Department of Pediatrics, All India Institute of Medical Sciences, New Delhi 110029, India; aiims.deepika@gmail.com; 7 Department of Pediatric Surgery, Jawaharlal Institute of Postgraduate Medical Education and Research, Puducherry 605006, India; drvjindal@gmail.com

**Keywords:** appendicitis, complicated appendicitis, biomarker, ischemia-modified albumin, systematic review, meta-analysis

## Abstract

Background: Acute appendicitis is a frequently encountered surgical emergency. Despite several scoring systems, the possibility of delayed diagnosis persists. In addition, a delayed diagnosis leads to an increased risk of complicated appendicitis. Hence, there is a need to identify biological markers to help clinicians rapidly and accurately diagnose and prognosticate acute appendicitis with a high sensitivity and specificity. Although several markers have been evaluated, the pressing concern is still the low specificity of these markers. One such marker is serum ischemia-modified albumin (IMA), which can be a novel biomarker for accurately diagnosing and prognosticating acute appendicitis. Methods: The authors conducted a systematic search of the PubMed, EMBASE, Web of Science, and Scopus databases through February 2023 as per the PRISMA guidelines. The difference in the levels of IMA between patients with acute appendicitis vs. healthy controls, and the difference in the levels of IMA between patients with complicated vs. non-complicated acute appendicitis were taken as the outcome measures. Statistical analysis was performed using a random effects model and mean difference (MD) was calculated. The methodological quality of the studies was assessed by utilizing the Newcastle–Ottawa scale. Results: A total of six prospective comparative studies were included in the meta-analysis. The analysis revealed that the mean level of serum IMA was significantly raised in the acute appendicitis group (MD 0.21, 95% CI 0.05 to 0.37, *p* = 0.01). Similarly, the mean serum IMA levels were also raised in the complicated appendicitis group compared to the non-complicated appendicitis group (MD 0.05, 95% CI 0.01 to 0.10, *p* = 0.02). Three of the studies included were, however, of poor methodological quality. Conclusions: Serum IMA is a viable potential marker for diagnosing and prognosticating acute appendicitis. However, due to the limited methodological quality of available studies, further prospectively designed and adequately powered studies are needed.

## 1. Introduction

Acute appendicitis is one of the most encountered surgical emergencies. Although several scoring systems such as the Alvarado score, the pediatric appendicitis score (PAS) and the pediatric appendicitis risk calculator (pARC) are available for diagnosing appendicitis in these patients, there is still a possibility of delayed diagnosis in these patients [1,2]. Moreover, it has also been shown that these scores have low sensitivity and specificity, with the Alvarado score having a sensitivity and specificity of only 54% and 75%, respectively. Conversely, although PAS and pARC both have good sensitivity (>90%), their specificity, however, is still around 50% [3,4]. As a result of this low specificity, up to 6% of the patients with suspected appendicitis may have a normal appendix on histopathology [5].

Furthermore, the rate of perforated or complicated appendicitis increases as the age of the patient decreases, with patients aged less than 5 years having perforation rates above 50% [6,7] and with these rates dropping down to around 25% in the pre-pubertal age (5–12 years) [8]. This, coupled with the low sensitivity and specificity of the available diagnostic criteria, underscores the fact that there is a need for the identification of biomarkers that can help clinicians in accurately diagnosing and prognosticating acute appendicitis with high sensitivity and specificity. Several markers with different sensitivity and specificity rates, e.g., including C-reactive protein (CRP), mean platelet volume (MPV), white blood cell (WBC) count, ESR and red cell distribution width (RDW), etc., have been explored for the diagnosis of acute appendicitis [9,10,11,12,13]. In addition, newer biomarkers, e.g., pentraxin-3, interleukin-6 (IL-6), calprotectin, serum bilirubin, leucine-rich-alpha-2-glycoprotein (LRG), and 5-hydroxyindoleacetic acid (5-HIAA), etc., have also been explored by some authors for an early diagnosis and predicting the outcomes in these patients [14,15,16,17,18,19,20]. 

With more and more research on biomarkers for appendicitis, it is clear that none of the available biomarkers is sacrosanct and has enough discriminatory ability to be used alone for diagnostic and prognostic purposes. Thus, the search for ‘an ideal biomarker’ is still ongoing. Some studies have recently evaluated the role of serum ischemia-modified albumin (IMA) as a potential marker for diagnosing acute appendicitis, differentiating it from other causes of abdominal pain, and as a marker to differentiate the complicated cases of appendicitis from the non-complicated ones. IMA is a surrogate marker of tissue ischemia, and its levels increase during the oxidative stress and inflammation occurring in acute appendicitis [5].

This study aims to systematically summarize and filter the available data to evaluate the usefulness of serum IMA in diagnosing and prognosticating patients with acute appendicitis. To our knowledge, this is the first systematic review and meta-analysis on this subject.

## 2. Materials and Methods

### 2.1. Protocol Registration

The present systematic review was registered in the international prospective register of systematic reviews (PROSPERO) on 5 February 2023 (CRD42023394470) [21]. 

### 2.2. Search Strategy

The systematic review and meta-analysis were performed in accordance with the Preferred Reporting Items for Systematic Reviews and Meta-Analyses (PRISMA) guidelines [22]. Two authors (SAn and AA) conducted a preliminary literature search in the PubMed database on 5 February 2023, identified the already published literature, and excluded the presence of existing meta-analysis on the topic of interest. Subsequently, a systematic literature search was conducted in the PubMed, EMBASE, Scopus, and Web of Science databases by both authors (Appendix A). The following key terms were utilized for performing the search: “Ischemia-modified albumin” [All Fields] AND (“appendicitis” [MeSH Terms] OR “appendicitis” [All Fields] OR (“acute” [All Fields] AND “appendicitis” [All Fields]) OR “acute appendicitis” [All Fields]). The duplicate records were removed and the remaining studies were screened per the eligibility criteria. 

### 2.3. Eligibility Criteria 

The inclusion criteria used were Participant—studies where patients (of any age) were diagnosed with acute appendicitis; Intervention—patients undergoing surgical or conservative management of acute appendicitis; Comparison—healthy controls, i.e., patients without any clinico-radiologic features of appendicitis; Outcomes—the difference in the levels of serum IMA between patients with acute appendicitis vs. healthy controls and the difference in the levels of serum IMA between patients with complicated (perforated or phlegmonous or gangrenous) vs. non-complicated acute appendicitis were taken as the outcome measures. Studies that reported at least one of the above-mentioned outcomes were included. No specific age criteria were applied and studies with adults and/or children were included. Non-comparative studies, case reports, editorials, letters to the editors, opinion articles, and conference abstracts were excluded. In addition, studies with unavailable full texts were also excluded. 

### 2.4. Data Synthesis 

Two investigators (DK and AS) independently performed the data extraction in Microsoft Excel (Version 2301) spreadsheets. Any disagreements among them were resolved through consensus or discussion with another investigator (ZP). Apart from the data on the above-mentioned outcomes, information regarding the name of the author, the year of publication, the type of study design, the number of patients assessed in each study, and the number of patients in each treatment group were extracted. 

### 2.5. Quality Assessment 

As the included studies were non-randomized, two investigators (CMLM and AV) independently assessed the methodological quality of the included studies via the validated Newcastle–Ottawa scale (NOS) [23]. 

### 2.6. Statistical Analysis

The analysis was performed by two authors (BJ and SAn). The baseline data were represented as numbers, proportions, averages, and ranges. The meta-analysis was conducted using RevMan 5.4 (Cochrane Collaboration, London, UK). As both the outcomes were continuous variables, the mean difference (MD) with a 95% CI was estimated using the nverse variance (IV) method. The random effects model was chosen for analysis since the studies had a varied methodology. The level of heterogeneity among the included studies was evaluated using the I^2^ statistics. A *p*-value of <0.05 was considered statistically significant.

## 3. Results

### 3.1. Study Characteristics 

**Figure 1:**
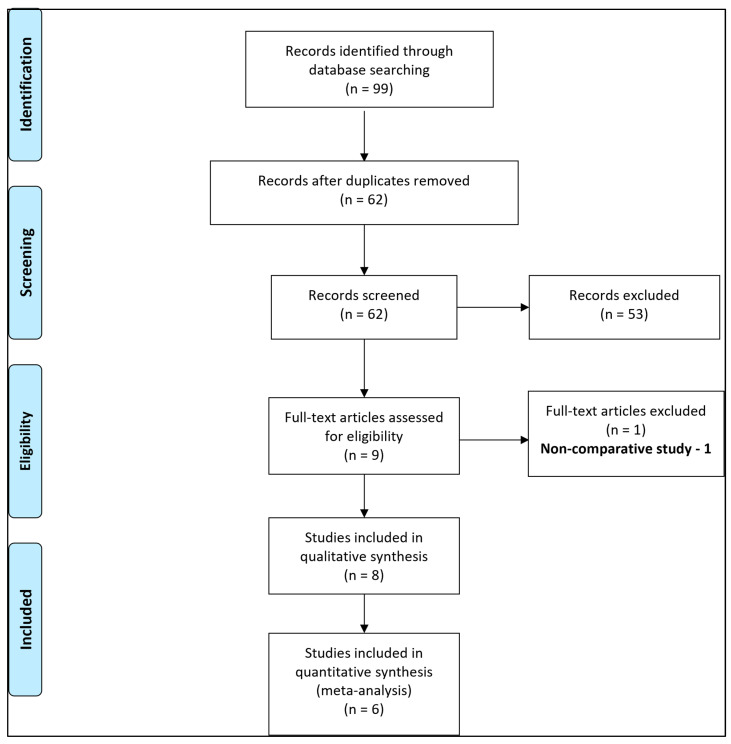
Selection of the studies using the Preferred Reporting Items for Systematic reviews and Meta-Analyses (PRISMA) flow diagram.

Out of 99 records identified with our search strategy (Appendix A), 37 duplicate articles were removed. The remaining 62 articles were screened for eligibility (Appendix A). Of these, 53 abstracts were excluded, and only nine full texts were assessed for inclusion (Figure 1). One of them was a non-comparative study and was excluded [24]. The remaining eight studies were included in the systematic review [5,25,26,27,28,29,30,31]. Of these, two studies [25,26] had used ng/mL as the unit of measurement of IMA and had not provided the specifications of measurement; thus, these values could not be converted to absorbance units (AbsU). Therefore, only six studies were included in the final meta-analysis [5,27,28,29,30,31]. The study designs of all these studies were prospective in nature. Four of these studies had included both the outcome measures [5,29,30,31], while the study by Nazik et al. [28] and Kılıç et al. [27] had included only the acute appendicitis group vs. control group, and complicated acute appendicitis group vs. non-complicated acute appendicitis group, respectively. The baseline characteristics of the studies included in the meta-analysis are demonstrated in Table 1.

### 3.2. Summary of the Studies Included in Meta-Analysis

#### 3.2.1. Dumlu et al., 2014 [5] 

This prospective comparative study from Turkey evaluated the serum and tissue levels of oxidative stress markers, including IMA, in patients with acute appendicitis with those of normal controls and between patients with complicated acute appendicitis (perforated and/or phlegmonous) and non-complicated acute appendicitis. The study included 30 controls and 65 patients with acute appendicitis. Appendectomy was performed in these 65 patients. Of the resected appendix specimens, 37 had non-complicated acute appendicitis, 24 patients had complicated appendicitis, and 4 showed no appendicitis. The serum IMA levels of the appendectomized patients were significantly elevated (0.64 +/− 0.09 vs. 0.31 +/− 0.09), *p* < 0.001) when compared with the control group. However, there was no significant difference (*p* = 0.337) between the complicated [perforated appendicitis (0.67 +/− 0.09), phlegmonous appendicitis (0.069 +/− 0.09), and perforated + phlegmonous appendicitis (0.67 +/− 0.1)] vs. the non-complicated appendicitis group (0.64 +/− 0.09). 

#### 3.2.2. Kılıç et al., 2017 [27] 

This study was also published in Turkey. The authors aimed to evaluate the effectiveness of serum IMA as a marker of appendiceal perforation. Of the 62 patients in this cohort study, 33 had non-complicated acute appendicitis while the remaining 29 had complicated acute appendicitis in gangrene or perforation. The serum IMA levels in the complicated acute appendicitis patients (0.682 +/−0.08) were found to be significantly elevated (*p* = 0.012) as compared to the non-complicated acute appendicitis group (0.618 +/− 0.09). Moreover, the authors also demonstrated a positive correlation (Spearman’s rho = +0.688, *p* = 0.003) between the raised serum IMA levels and the reporting of complications on computed tomography (CT). 

#### 3.2.3. Nazik et al., 2017 [28]

This prospective study was conducted in Turkey and compared the serum level of IMA and other inflammatory markers in acute appendicitis patients with healthy controls. Of the 63 patients that constituted the cohort, 30 were cases of acute appendicitis, while the remaining 33 were healthy controls. The study demonstrated that the serum levels of IMA in patients with acute appendicitis (0.56 +/− 0.1) were significantly raised (*p* < 0.001) when compared to those of healthy controls (0.33 +/− 0.1). Similarly, the erythrocyte sedimentation rate (ESR), c-reactive protein (CRP), white blood cell count, neutrophil-lymphocyte ratio, and platelet-lymphocyte ratio were also significantly raised in patients with acute appendicitis. On a receiver operating characteristic (ROC) curve, IMA was found to have the highest area under the curve (0.991), suggesting its potential as a diagnostic marker for acute appendicitis. 

#### 3.2.4. Sarac et al., 2019 [29] 

Again published in Turkey in 2019, this prospective comparative study evaluated the diagnostic value of IMA in patients with acute abdomen. Noteworthy in this study was that apart from patients with acute appendicitis and healthy controls, they had also included patients with non-specific abdominal pain (NSAP). Of the total patient population of 152 patients, 54 were diagnosed cases of non-complicated acute appendicitis, 16 had complicated (perforated) acute appendicitis, 42 were patients with NSAP, while the remaining 40 were controls. They had hence shown that the serum levels of IMA in the patients with the non-complicated acute appendicitis group were significantly elevated as compared to those of NSAP (0.70 +/− 0.17 vs. 0.46 +/− 0.17, *p* < 0.001) as well as controls (0.70 +/− 0.17 vs. 0.36 +/− 0.08, *p* < 0.001). However, there was no significant difference in the serum IMA levels of these patients when compared with that of complicated acute appendicitis (0.73 +/− 0.17) (*p* = 0.576).

#### 3.2.5. Ünsal et al., 2022 [30]

The authors conducted this prospective study in Turkey, which is the most recent study of this meta-analysis. They enrolled 139 patients, of whom 42 were controls and 97 were in the acute appendicitis group. The mean serum IMA levels in the acute appendicitis group were 0.77 +/− 0.14, significantly higher (*p* = 0.001) as compared to the control group (0.693 +/− 0.16). They further subdivided the acute appendicitis patients into those with non-complicated acute appendicitis (*n* = 64), acute suppurative appendicitis (*n* = 13), and perforated appendicitis (*n* = 20). In a sub-analysis, the authors demonstrated that within the patients with acute appendicitis, the mean serum IMA levels were not statistically different (*p* = 0.234), with the levels being 0.78 +/− 0.12 in patients with non-complicated acute appendicitis and 0.78 +/− 0.14 and 0.78 +/− 0.12 in the patients with acute suppurative appendicitis and perforated appendicitis, respectively. 

#### 3.2.6. Hakkoymaz et al., 2019 [31] 

This was also a comparative study conducted in Turkey and demonstrated the usefulness of serum IMA levels in the diagnosis of acute appendicitis along with other oxidative stress markers (malondialdehyde and glutathione peroxide). This study was also in line with the previous studies and showed that in the patients with acute appendicitis (*n* = 51), the serum IMA levels were significantly (*p* < 0.001) elevated (0.33 +/− 0.1) when compared with the control (*n* = 45) population (0.21 +/− 0.1). Also, the serum IMA levels were again significantly elevated in patients with complicated acute appendicitis as compared to patients with non-complicated acute appendicitis (0.40 +/− 0.05 vs. 0.29 +/− 0.04, *p* < 0.001). 

### 3.3. Quality Assessment 

Upon methodological assessment using the Newcastle–Ottawa scale (Table 2), only three of the included studies were of good quality [28,29,31], while the remaining three were of poor methodological quality and were also weak in the comparability domain [5,27,30]. 

### 3.4. Meta-Analysis

(a) Serum IMA in patients with acute appendicitis vs. controls

**Figure 2:**
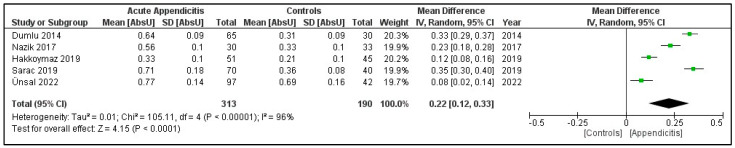
Forest plot comparison of serum IMA levels among patients with acute appendicitis vs. controls [5,28,29,30,31].

A total of five studies were included in this analysis (Figure 2), with 313 patients in the acute appendicitis group and 190 patients in the control group. The mean difference between both the groups was statistically significant, with the mean serum IMA level being raised in the acute appendicitis group (MD 0.22, 95% CI 0.12 to 0.33, *p* ≤ 0.0001). The heterogeneity of the included studies was, however, significantly substantial (I^2^ = 96%, *p* < 0.00001).

(b) Serum IMA in patients with complicated vs. non-complicated acute appendicitis

**Figure 3:**
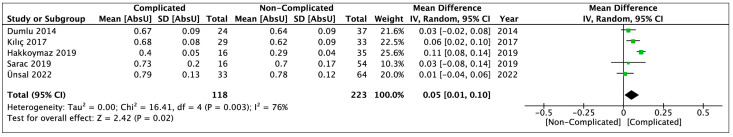
Forest plot comparison of serum IMA levels among patients with complicated acute appendicitis vs. non-complicated acute appendicitis [5,27,29,30,31].

The five studies included 118 and 223 patients in complicated and non-complicated appendicitis groups, respectively (Figure 3). The mean difference of the serum IMA levels between both complicated appendicitis and non-complicated appendicitis groups was statistically significant (MD 0.05, 95% CI 0.01 to 0.10, *p* = 0.02); however, there was a presence of substantial heterogeneity among the included studies (I^2^ = 76%, *p* = 0.003).

## 4. Discussion

Acute appendicitis is a frequently encountered surgical condition in emergency departments. The treating physicians rely on a combination of factors such as clinical history, physical examination, laboratory tests, and radiological findings to identify patients at a higher risk of appendicitis. Despite this, there is still a possibility of delayed diagnosis, misdiagnosis, and unnecessary surgery, particularly among younger children with atypical symptoms. Multiple clinical scoring systems have been developed for diagnosing appendicitis [32]. These include the Alvarado score, the pediatric appendicitis score (PAS) and the pediatric appendicitis risk calculator (pARC) [1,2]. These scores help classify patients into low, moderate, or high-risk groups for appendicitis. However, their ability to determine which patients require appendectomy is limited. Hence, current research focuses on identifying inflammatory markers that can be used as a definitive method for diagnosing and prognosticating acute appendicitis. The most frequently evaluated marker is CRP, which has been reported in studies to have a sensitivity and specificity of 100% and >80%, respectively [9]. Other markers that have been evaluated include mean platelet volume (MPV), white blood cell (WBC) count, ESR, red cell distribution width (RDW), Pentraxin-3, hyponatremia, and oxidative markers such as nitric oxide and myeloperoxidase, to name a few [10,11,12,13,33,34,35,36,37].

IMA is a recently studied marker and is considered as a surrogate of local tissue ischemia in various conditions [38]. It has been observed that under oxidative stress, due to the generation of reactive oxygen species and tissue acidosis, the N-terminal of the serum album undergoes degradation resulting in a reduction in its affinity to transition metals, especially cobalt. This can occur either due to dipeptide cleavage of albumin or the removal of the few N-terminal amino acids caused by increased free radicals [39,40]. This variant of human albumin with low metal binding capacity is known as IMA. Another theory of the formation of IMA states that due to ischemia (especially myocardial ischemia), fatty acids are released, which bind with albumin, resulting in a decreased affinity for cobalt [41]. IMA is a transient and reversible variant which reverts to normal albumin once the stress subsides. Studies have shown that the serum IMA levels start rising within 6–10 min of the insult and remain elevated for 6–12 h, with the baseline levels being attained in 12–24 h; these kinetics were, however, demonstrated in patients with myocardial ischemia in which IMA has been prevalently studied [42,43]. Also, it has been shown that the duration of ischemia also affects the levels of IMA in serum, with prolonged ischemia resulting in increased levels. The pathophysiological process of appendicitis leads to mucosal obstruction and local ischemia, which can present with increased IMA levels in the serum. 

Several studies [5,25,26,27,28,29,30,31] have compared the levels of serum IMA in patients with acute appendicitis to those of controls and other causes of abdominal pain. Ulusoy et al. [25], have demonstrated significantly higher levels of IMA in appendicitis vs. no appendicitis, and appendicitis vs. controls. In a sub-analysis, the authors also demonstrated significantly lower levels of IMA in patients with a negative appendectomy vs. those with histopathological features of appendicitis. Yeniocak et al. [26], compared the serum IMA levels of patients with non-traumatic acute abdominal pain (n = 194) vs. controls (n = 140). The authors also had similar findings of significantly elevated levels of IMA in patients with acute appendicitis vs. controls.

However, to our knowledge, this study is the first systematic review and meta-analysis on this subject. The analysis showed that patients with acute appendicitis have significantly (*p* < 0.0001) elevated serum IMA compared to the control group. Moreover, the patients with complicated appendicitis, i.e., with perforated and/or phlegmonous appendicitis, also had a significantly higher (*p* = 0.02) serum concentration of IMA when compared with those of non-complicated appendicitis. Most of the included studies, however, had a mix of both adult and pediatric populations, with only two of the studies focusing primarily on pediatric (<18 years) patients [28,29]. These findings align with the expected pathophysiological processes, the observed increase in other acute phase reactants, and the markers of oxidative stress in these patients. 

When compared to other established biochemical markers that have been evaluated for appendicitis, such as WBC count, CRP, procalcitonin, MPV, and neutrophil-lymphocyte ratio (NLR), IMA has been shown to have high sensitivity (>95%) and specificity (>70%), with a high negative predictive value of >95% [28,29]. This makes IMA a better biomarker as compared to the other parameters. Moreover, with the availability of more non-invasive biomarkers such as salivary leucine-rich-alpha-2-glycoprotein [18], the focus should be on developing a panel of minimally invasive highly sensitive and specific biomarkers coupled with clinical-radiological findings to make the diagnosis and prognostication more rapid and accurate. 

A few limitations to the current study are to be noted while interpreting the results. First, the risk of bias in three of the included studies was high. Moreover, the sample size of the given studies is small, adding to the bias. Also, since most studies have a varied population distribution by age, the results are further confounded. Second, since IMA is a non-specific marker of tissue ischemia, it can rise in various conditions and may overpredict the results if used as ‘a single biomarker’. Third, it is important to note that serum IMA levels can be altered due to changes in the serum albumin levels. Finally, the studies have used a non-standardized method of differentiating the patients into complicated and non-complicated appendicitis, which can also add to the bias of the included studies. Hence, it is imperative to note that future studies need to be designed to address the above limitations, assess these biomarkers as a panel, and further augment them with clinical and radiological criteria. 

## 5. Conclusions

Serum IMA levels can act as a potential marker of diagnosis of acute appendicitis as they are significantly raised in these patients compared to controls. Moreover, they can also act as a possible prognostication factor as they are also significantly raised in patients with complicated appendicitis. However, due to the limited methodological quality of the included studies, future prospective, adequately powered studies need to be conducted before any definite conclusions in this regard are drawn.

## Figures and Tables

**Table 1:** Basic characteristics of the studies included in the analysis.

Author/Year	Groups	N	Age	Male/ Female	TLC (per µL)	CRP (mg/dL)	MPV (fL)	Serum IMA Levels (AbsU)
Dulmu 2014 [5]	Control	30	-	-	-	-	-	0.31 +/− 0.09
	No Appendicitis	4	30.5 +/− 11.1 (Years)	1/3	9650 +/− 2154.54	-	-	0.65 +/− 0.09
	Non-Complicated Acute Appendicitis	37	32.06 +/− 10.47 (Years)	15/22	12,021.63 +/− 4751.62	-	-	0.64 +/− 0.09
	Perforated Appendicitis	8	34.88 +/− 15.74 (Years)	5/3	12,737.5 +/− 3601.17	-	-	0.67 +/− 0.09
	Phlegmonous Appendicitis	12	25.92 +/− 6.49 (Years)	8/4	11,583.34 +/− 4683.41	-	-	0.67 +/− 0.09
	Perforated + Phlegmonous Appendicitis	4	33.5 +/− 9.82 (Years)	1/3	14,475 +/− 8109.82	-	-	0.67 +/− 0.10
Kılıç 2017 [27]	Non-Complicated Acute Appendicitis	33	29.8 +/− 10.7 (Years)	20/13	15,100 +/− 3400	-	-	0.618 +/− 0.09
	Gangrenous/Perforated Appendicitis	29	30.4 +/− 11.3 (Years)	13/16	15,200 +/− 5900	-	-	0.682 +/− 0.08
Nazik 2017 [28]	Control	33	105.6 +/− 30.8 (Months)	23/10	7730 +/− 2100	7.45 +/− 9.2	8.08 +/− 0.9	0.33 +/− 0.1
	Acute Appendicitis	30	119 +/− 27.4 (Months)	18/12	12,120 +/− 4800	29.63 +/− 41.3	8.23 +/− 0.8	0.56 +/− 0.1
Hakkoymaz 2019 [31]	Control	45	30.9 +/− 12.3 (Years)	29/16	7700 +/− 2100	3.7 +/− 1.6	10.4 +/− 0.9	0.21 +/− 0.1
	Non-Complicated Acute Appendicitis	35	33.6 +/− 16.2 (Years)	29/22	12,400 +/− 4500	25.4 +/− 32.1	9.6 +/− 2.2	0.29 +/− 0.04
	Complicated Appendicitis	16			12,200 +/− 4700	43.8 +/− 52.2		0.40 +/− 0.05
Sarac 2019 [29]	Control	40	8.4 +/− 4.8 (Years)	26/16	-	-	-	0.36 +/− 0.08
	Non-Complicated Acute Appendicitis	54	10.2 +/− 3.3 (Years)	36/17	15,040.0 +/− 3688.7	2.34 +/− 5.89	-	0.70 +/− 0.17
	Perforated Appendicitis	16	9.3 +/− 3.0 (Years)	8/8	18,241.3 +/− 4865.1	7.62 +/− 9.47	-	0.73 +/− 0.20
Unsal 2022 [30]	Control	42	35.93 +/− 10.07 (Years)	24/18	9320 +/− 3610	5.860 +/− 6.224	-	0.693 +/− 0.16
	Non-Complicated Acute Appendicitis	64	35.75 +/− 12.13 (Years)	53/44	13,630 +/− 3920	5.470 +/− 5.531	-	0.78 +/− 0.12
	Acute Suppurative Appendicitis	13	-		14,200 +/− 5440	6.675 +/− 6.076	-	0.78 +/− 0.14
	Perforated Appendicitis	20	-		16,010 +/− 4450	9.269 +/− 7.612	-	0.79 +/− 0.12

Abbreviations: TLC, total leukocyte count; CRP, C-reactive protein; MPV, mean platelet volume; IMA, ischemia-modified albumin; µL, microlitre; mg, milligram; dl, decilitre; fL, femtoliters; AbsU, absorbance unit.

**Table 2:** Methodological quality assessment utilizing the Newcastle–Ottawa scale.

Author/ Year	Selection			Comparability			Outcomes		Total Score	Quality ^#^
	Item 1	Item 2	Item 3	Item 4	Item 5	Item 6	Item 7	Item 8		
Dulmu 2014 [5]	*	*	*	*	-	*	*	*	7	Poor
Kılıç 2017 [27]	*	-	*	*	-	*	*	*	6	Poor
Nazik 2017 [28]	*	*	*	*	*	*	*	*	8	Good
Hakkoymaz 2019 [31]	*	*	*	*	**	*	*	*	9	Good
Sarac 2019 [29]	*	*	*	*	*	*	*	*	8	Good
Unsal 2022 [30]	*	*	*	*	-	*	*	*	7	Poor

^#^ Good Quality: 3 or 4 stars in selection domain AND 1 or 2 stars in the comparability domain AND 2 or 3 stars in the outcome domain. Poor Quality: 0 or 1 star(s) in selection domain OR 0 stars in the comparability domain OR 0 or 1 star(s) in the outcome domain.

## Data Availability

Data that support this study are available from the corresponding author upon reasonable request.

## Appendix A

PubMed—(‘ischemia modified albumin’) AND (appendicitis)
Embase—All Fields: ‘ischemia modified albumin’ AND ‘appendicitis’
Scopus—ALL (“ischemia modified albumin”) AND ALL (“appendicitis”)
Web of Science—(ALL = (ischemia modified albumin)) AND ALL = (appendicitis)

**Table A1:** Results of the systematic literature search in various databases.

Database	Studies
PubMed	9
EMBASE	11
Web of Science	10
Scopus	69
Total	99
Duplications	37
After duplication removal	62
